# Transcriptional Evidence for Inferred Pattern of Pollen Tube-Stigma Metabolic Coupling during Pollination

**DOI:** 10.1371/journal.pone.0107046

**Published:** 2014-09-12

**Authors:** Xun Yue, Xin-Qi Gao, Fang Wang, YuXiu Dong, XingGuo Li, Xian Sheng Zhang

**Affiliations:** 1 State Key Laboratory of Crop Biology, College of Life Sciences, Shandong Agricultural University, Tai'an, Shandong, China; 2 College of Information Sciences and Engineering, Shandong Agricultural University, Taian, Shandong, China; National Institute of Genomic Medicine, Mexico

## Abstract

It is difficult to derive all qualitative proteomic and metabolomic experimental data in male (pollen tube) and female (pistil) reproductive tissues during pollination because of the limited sensitivity of current technology. In this study, genome-scale enzyme correlation network models for plants (*Arabidopsis*/maize) were constructed by analyzing the enzymes and metabolic routes from a global perspective. Then, we developed a data-driven computational pipeline using the “guilt by association” principle to analyze the transcriptional coexpression profiles of enzymatic genes in the consecutive steps for metabolic routes in the fast-growing pollen tube and stigma during pollination. The analysis identified an inferred pattern of pollen tube-stigma ethanol coupling. When the pollen tube elongates in the transmitting tissue (TT) of the pistil, this elongation triggers the mobilization of energy from glycolysis in the TT cells of the pistil. Energy-rich metabolites (ethanol) are secreted that can be taken up by the pollen tube, where these metabolites are incorporated into the pollen tube's tricarboxylic acid (TCA) cycle, which leads to enhanced ATP production for facilitating pollen tube growth. In addition, our analysis also provided evidence for the cooperation of kaempferol, dTDP-alpha-L-rhamnose and cell-wall-related proteins; phosphatidic-acid-mediated Ca^2+^ oscillations and cytoskeleton; and glutamate degradation IV for γ-aminobutyric acid (GABA) signaling activation in *Arabidopsis* and maize stigmas to provide the signals and materials required for pollen tube tip growth. In particular, the “guilt by association” computational pipeline and the genome-scale enzyme correlation network models (GECN) developed in this study was initiated with experimental “omics” data, followed by data analysis and data integration to determine correlations, and could provide a new platform to assist inachieving a deeper understanding of the co-regulation and inter-regulation model in plant research.

## Introduction

The communication between the male (pollen) and female (pistil) reproductive tissues remains an exciting frontier in pollination/fertilization research [1]. Compatible pollen–stigma interactions are crucial steps for the success of seed production in flowering plants, identifying and interpreting the systemic mechanisms of pollen–stigma interactions may benefit for positive impact on productivity in crop plants [2]. Over the past two decades, significant progress has been made in elucidating the molecular events of the pollen–stigma interaction in self-incompatible (SI) pollination systems; however, the detailed molecular mechanisms of the interaction between female and male reproductive tissues in compatible pollination are still unclear [3]. Experimental data have demonstrated that pollen tubes can germinate and elongate in a synthetic medium, that the trajectory of pollen tubes is random, and that the growth rate of pollen tubes is lower *in vitro* compared with *in vivo* [4]. Genetic and genomic approaches have shown that initial pollen tube growth is autotrophic, and uses the stored nutrients in pollen grains, whereas for the subsequent growth, heterotrophic pollen tube absorbs and metabolizes external energy-rich metabolites for the generation of energy (ATP) and the construction of new cell walls [5], [6], [7]. To maintain the high growth rate of the growing pollen tube, protein biosynthesis and metabolic pathways in the male (pollen) and female (pistil) reproductive tissues are activated to provide the signal, materials and energy for facilitating pollen germination and tube growth. However, our understanding of the mechanisms of pollen tube-stigma metabolic coupling for manipulating pollen tube growth during the pollen–stigma interaction remains incomplete.

The “omics” approaches, such as transcriptomics and proteomics, have been used to elucidate the molecular mechanisms and the signaling pathways during the pollen–stigma interaction [8]–[22]. The challenge is how to use these rich “omics” data sets to elucidate meaningful regulatory relationships between mRNAs, proteins and other biological molecules [23]. Large networks of intermolecular interactions are being measured systematically for humans and many model species. Such networks include physical associations underlying protein-protein, protein-DNA or metabolic pathways. Numerous approaches have been developed to mine such networks for identifying biological modules. They have been extremely powerful for elucidating molecular machineries [24], [25]. Recently, several genome-scale models of metabolic network reconstructions have become available for plants [26], [27], [28]. In particular, the metabolic pathway networks of *Arabidopsis* and maize have been constructed from “omics”-scale data related to pollen development and pollen tube growth [7], [29]. In lily pollen (*Lilium longiflorum*), Gerhard et al. [7] investigated the metabolome of lily pollen *in vitro* and provided a comprehensive overview of the metabolites active during pollen germination and tube growth. However, there is not the proteomic and metabolomic experimental dataset available for *Arabidopsis* or maize about the pollen-stigma communication. Because of the difficulties in deriving qualitative or, at best, semi quantitative proteomic and metabolomic experimental data related to male (pollen tube) and female (pistil) reproductive tissues, genome-wide transcriptome analyses for genes that encode enzymes, which use microarray and RNA-seq technology, could provide an ideal starting point for analyzing the global perspective of the active enzymes and metabolic routes in fast-growing pollen tubes and stigmas during pollination. However, the transcriptional level change is only one part of the regulatory process; factors such as RNA stability, translation rates, protein processing and stability, and metabolite concentrations also play essential roles in the fine-scale moderation of cellular activity. Therefore, changes at the transcriptional level might not equate to differences at the protein level and certainly not at the enzyme activity level [30], [31]. However, studies have shown that correlated genes are likely to be involved in similar (or identical) metabolism pathways in higher plants [32], [33]. Particularly, Wei et al. [34] performed a comprehensive analysis of the coexpression networks of 1330 genes from the AraCyc database of metabolic pathways in *Arabidopsis*. These authors found that the genes associated with the same metabolic pathway are, on average, more highly coexpressed compared with the genes from different pathways. If the transcriptional level genes that encoded the co-expressed enzymes in the consecutive steps of metabolic routes were significantly different at different time points during the pollen–stigma interaction, then it is conceivable that these metabolic routes could be critical during the pollen–stigma interaction. Therefore, the transcriptiona coexpression profiles of enzymatic genes in the consecutive steps for metabolic routes can reveal the functional status of the biological process. We designated this data-driven computational pipeline using a concept from the court of law, “guilt by association”, which denotes that the defendant was present at the scene of the crime [35].

In this study, we developed the “guilt by association” computational pipeline and the genome-scale enzyme correlation network models (GECN) to analyze the global perspective of the active enzymes and metabolic routes in fast-growing pollen tubes and in stigmas during pollination. First, we identified the significant differentially expressed genes (DEGs) of the pollen tube and the stigmas in response to pollination. Second, the significant DEGs that encoded enzymes were mapped to the GECN model, and a sub-interaction network was constructed. We determined that the genes that encoded co-expressed enzymes were involved in consecutive steps for several specific metabolic routes. Therefore, these metabolic routes can reveal the functional status during pollination.

## Materials and Methods

### Materials and data

#### (i) Maize stigmas

The maize inbred line Zheng58 was grown in the field of the Shandong Agricultural University Experimental Station, Tai'an, China. Maize silk was collected as mature silk, silk at 20 min after pollination, and silk at 3 h after pollination. Within the first 20 min after pollination, most pollen grains hydrated and germinated on the silk hairs, and invaded the stigmatic tissues. At 3 h after pollination, pollen tubes were growing inside the transmitting tracts. The experiment was performed using three biological replicates for each time point. Total RNA was extracted according to the modified CTAB protocol and purified using an RNeasy MinElute Cleanup Kit (Qiagen, Valencia, CA, USA). The RNA concentration and purity was quantified by Nanodrop spectrophotometry (Nanodrop Technologies, Wilmington, DE, USA), and integrity was confirmed by an Agilent 2100 Bioanalyzer (Agilent Technologies, Palo Alto, CA, USA) [22]. After extracting the total RNA from the samples, the total RNA was enriched using oligo (dT) magnetic beads. Fragmentation buffer was added to obtain short mRNA fragments (approximately 200 bp). Then, the first strand cDNA was synthesized using a random hexamer-primer and the mRNA fragments as templates dNTPs, RNase H and DNA polymerase I were added to synthesize the second strand. The double-strand cDNA was purified with a QiaQuick PCR extraction kit and washed for end repair and single nucleotide A (adenine) addition. Finally, sequencing adaptors were ligated to the fragments. The required fragments were purified by agarose gel electrophoresis and enriched by PCR amplification. The library products were sequenced using the Illumina HiSeq 2000. The original image data were transformed into sequence data by base calling, and the raw reads that were obtained were saved as FASTQ files. The raw reads were cleaned for data analysis. The gene expression levels were calculated using the RPKM method (reads per kilobase of transcript per million reads). The raw data sets (CEL) and the normalized expression data sets were deposited in the EBI ArrayExpress database (ArrayExpress: E-MTAB-964).

#### (ii) *Arabidopsis* stigmas

We used the *Arabidopsis* Affymetrix ATH1 oligonucleotide microarray data that were published by Boavida, who used the ATH1 whole genome array to compare the profiles of wild-type unpollinated pistils with the expression profile of pistils at 0.5, 3.5 and 8.0 h after pollination [20]. In *Arabidopsis*, within the 0.5 hour after pollination (HAP), most pollen grains had hydrated, germinated, and invaded the stigmatic papilla cells. At 3.5 HAP, the pollen tubes had grown through the style TT cells, and at 8.0 HAP, few pollen tubes were in their final guidance stages to, or interacting with, the embryo sac; however, most ovules were fertilized (Series accession number GSE27281).

#### (iii) *Arabidopsis* pollen tube response to pollination

In *Arabidopsis*, pollen tubes grow deep within a solid style; thus, it is extremely difficult to obtain sufficient quantities of pure *in vivo*-grown pollen tubes for microarray analysis. Qin et al. [18] overcame this challenge by collecting many pollen tubes that had grown through pistil tissue using a semi-*in vivo* method. In this paper, we used their comparative microarray data from dry, un-germinated pollen (dry pollen), pollen grown *in vitro* for 0.5 h (0.5 HPT) or for 4 h (4 HPT) and pollen germinated and grown through the stigma and style (SIV PT) (Series accession number GSE17343).

#### (iv) Metabolome of the pollen of lily (*Lilium longiflorum*) *in vitro*

Gerhard et al. [7] investigated the metabolome of lily pollen (Lilium longiflorum) *in vitro* and provided a comprehensive overview of the metabolites active during pollen germination and tube growth. More than 100 different metabolites were determined simultaneously by gas chromatography coupled with mass spectrometry. (European Nucleotide Archive, http://www.ebi.ac.uk/ena/data/view/ERP002303).

### Construction of the GECN model for *Arabidopsis* and maize

Source files were downloaded from a plant metabolic pathway database (PMN/PlantCyc) (http://www.plantcyc.org), from AraCyc 10. 0 (http://www.Arabidopsis.org/biocyc/), which is comprised 540 pathways, 7127 enzymes, 3418 reactions, 3323 compounds and 4225 citations, and from CornCyc 2.0 (http://pmn.plantcyc.org/organism-summary?object=CORN), which is comprised 373 pathways, 10527 enzymes, 2198 reactions, 1752 compounds and 2344 citations. We reconstructed the GECN model as follows:

**Step 1**: Source files (TXT file) were downloaded from AraCyc 10. 0 and CornCyc 2.0.

**Step 2**: Plant metabolic pathway data file (pathways, enzymes, genes, reactions, compounds and citations) were imported to the platform of Oracle Database.

**Figure 1:**
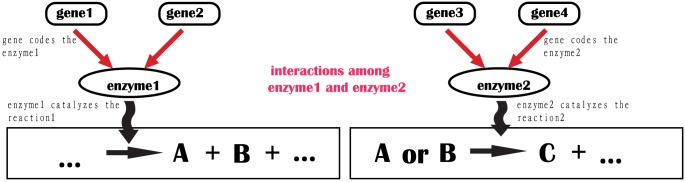
The interaction between two enzymes in genome-scale enzyme correlation network models. The reactions between metabolites were used to determine the interactions among enzymes. If enzyme 1 is catalyzed to produce substrates A and B, which are then used by enzyme 2 (substrate A or B produce C), the interaction was defined as enzyme 1 and enzymes 2.

**Step 3**: As depicted in Figure 1, the reactions between metabolites were used to determine the interactions among enzymes. In GECN models, enzymes are represented by nodes. If enzyme 1 is catalyzed to produce substrates A and B, which are then used by enzyme 2 (substrate A or B produce C), the interaction was defined as enzyme 1 and enzymes 2. The construction of the GECN was done in the platform of Oracle Database and Structured Query Language.

**Step 4**: Because small molecules—H^+^, ADH, NADP, NADPH, NH^3^, ATP, ADP, AMP, NAD, CoA, O^2^, CO^2^, Glu and pyrophosphate—are involved in many reactions, or are used as carriers for transferring electrons, they were excluded from the analysis in GECN models.

**Step 5**: GECN models for *Arabidopsis* and maize was visualized using Cytoscape software [16] (Data S1 and Data S2). This network models provide the new platform for analyzing the enzymes and metabolic routes from global perspective.

### Schematic of the “guilt by association” computational pipeline

**Figure 2:**
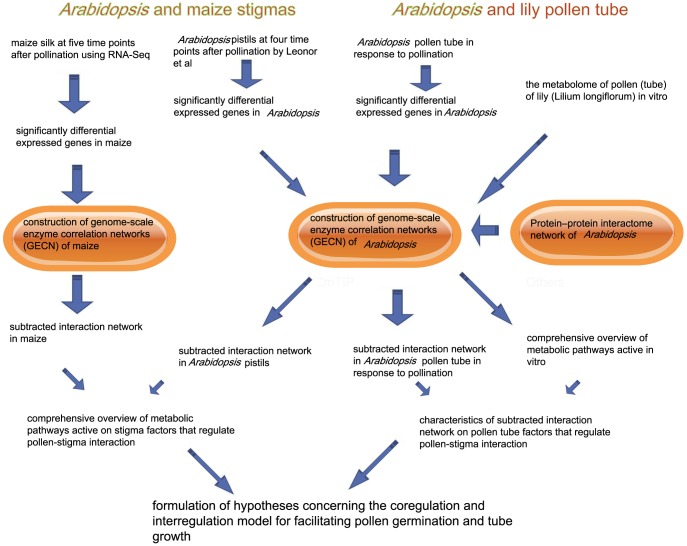
Schematic of “guilt by association” computational pipeline. Metabolome of the pollen (tube) of lily (Lilium longiflorum), the significant DEGs of the *Arabidopsis* pollen tube and the DEGs of *Arabidopsis* and of maize stigmas were identified in the platform of Oracle Database and SQL (Structured Query Language). Then, metabolome of the pollen (tube) of lily, the significant DEGs that encoded enzymes of *Arabidopsis* and of maize were mapped to the GECN model and the protein–protein interactome network of *Arabidopsis*, and a sub-interaction network was constructed by the Cytoscape software. Furthermore, community analyses (NetworkAnalyzer) were used to investigate the characteristics of the systemic structure of the sub-interaction network, and to analyze transcriptional levels of genes encoding co-expressed enzymes in the consecutive steps for metabolic routes in the biological process.

As shown in Figure 2, based on the constructed genome-scale enzyme correlation network (GECN) models and the proteome-wide binary protein–protein interaction map of *Arabidopsis*, we first used an *in vitro* metabolome of the pollen (tube) of lily (*Lilium longiflorum*) to provide a comprehensive overview of metabolic pathways that are active during pollen germination and during tube growth. Second, we identified the significant DEGs of the *Arabidopsis* pollen tube in response to pollination and the DEGs of *Arabidopsis* and of maize stigmas when the pollen tube elongated in the TT of the pistil compared with the stigmas in which pollination had not occurred. The significant DEGs that encoded the enzymes of *Arabidopsis* and of maize in response to pollination were done in the platform of Oracle Database and SQL (Structured Query Language). Then, the significant DEGs that encoded enzymes of *Arabidopsis* and of maize in response to pollination were mapped to the GECN model, and a sub-interaction network was constructed. Furthermore, a multi-omics network analysis and community analyses were used to investigate the characteristics of the systemic structure of the sub-interaction network, and to analyze transcriptional levels of genes encoding co-expressed enzymes in the consecutive steps for metabolic routes in the biological process. Lastly, the coregulation and interregulation model of the co-expressed enzymes in the consecutive steps for metabolic routes for facilitating pollen germination and tube growth is also discussd.

### Gene ontology analysis

A Cytoscape plugin, the biological networks gene ontology tool (BiNGO 2.3), was used to identify overrepresented gene ontology (GO) terms. Over-represented GO terms were selected using corrected p-values at significance levels of 5E-5 [36].

### Protein–protein interactome network of *Arabidopsis*

We downloaded and parsed the interaction information of a proteome-wide binary protein–protein interaction map of *Arabidopsis* from the *Arabidopsis* Interactome Mapping Consortium [37], which contains approximately 6,200 highly reliable interactions between approximately 2,700 proteins.

### Network topology analysis

NetworkAnalyzer is a Java plugin for Cytoscape [75]. This plugin computes specific parameters that describe the network topology [38]. In this study, we used NetworkAnalyzer to perform an analysis of the number of connected pairs of nodes to examine the overall structure of the GECN model.

## Results

### Significant DEGs of the *Arabidopsis* pollen tube and the *Arabidopsis* and maize stigmas in response to pollination

#### (i) Stigma response to pollination

Maize silks can be regarded as stigma, which are particularly extended and fused over most of their length [39]. Within the first 20 min after pollination, most pollen grains had hydrated and germinated on the silk hairs, and the pollen tubes had invaded the stigmatic tissues. At 3 HAP, the pollen tubes were growing inside the transmitting tracts. In our previous study, the transcriptomes of maize silk at 20 min and 3 HAP were analyzed using RNA-seq technology (described in the “Materials and Methods”) [22]. For *Arabidopsis*, we used the microarray data published by Boavida, who used the ATH1 whole genome array to compare the profiles of wild-type unpollinated *Arabidopsis* pistils with the profiles of pistils at 0.5, 3.5, and at 8.0 HAP [20]. In *Arabidopsis*, at 0.5 HAP, most pollen grains had hydrated, germinated, and invaded the stigma papilla cells. At 3.5 HAP, the pollen tubes were growing through the TT cells. At 8.0 HAP, most ovules were fertilized, and only a few pollen tubes were in their final guidance stages to the embryo sac.

Using a fold change ≥2 and a false discovery rate ≤1E-10 as the criteria, the genes that showed differential expression in at least one of the processes were regarded as DEGs. We identified 6,334 genes in maize silk and 1,648 genes in *Arabidopsis* pistil, with significant levels of differential expression (**Data S3**).

#### (ii) Pollen tube response to pollination

In *Arabidopsis*, pollen tubes grow deep within a solid style; thus, it is extremely difficult to obtain sufficient quantities of pure *in vivo*-grown pollen tubes for microarray analysis. In this paper, based on Qin et al.'s comparative microarray data, we identified 1,121 genes with significant DEGs of the *Arabidopsis* pollen tube between SIV PT and 4 HPT (Data S3).

Using the biological networks gene ontology tool (BiNGO 2.3) (FDR correction, significance level is 0.05) [36], the gene ontology enrichment analysis revealed that many DEGs were enriched in several classification categories, such as “metabolic process”, “biosynthetic process”, “cell wall modification”, “plant-type cell wall loosening”, and “defense response to bacterium” (Data S4).

### Overview of the metabolic routes active in the *Arabidopsis* pollen tube in response to pollination and in the *Arabidopsis* and maize stigmas during pollination

**Figure 3:**
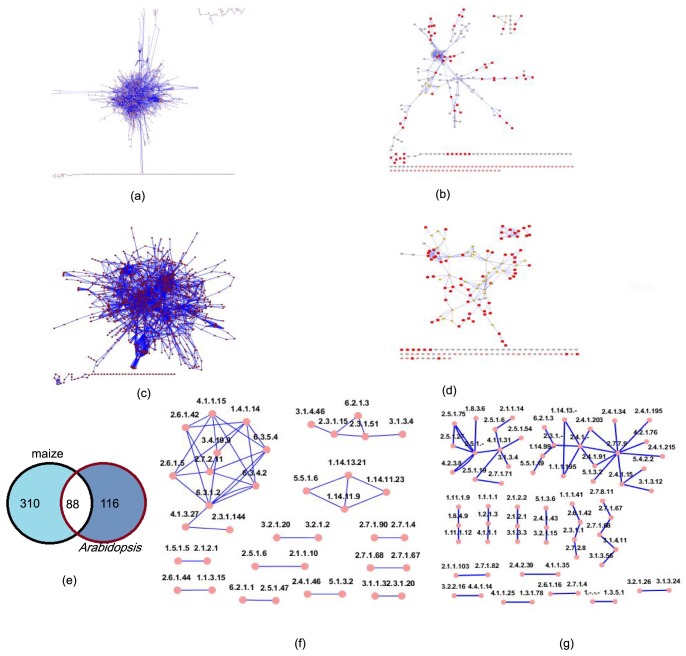
Sub-network of GECN. (a) Full GECN model of maize silk. SIF Cytoscape formation in Data S2. (b)Sub-interaction network of maize silk. .xlse file in Data S5. (c) Full GECN model of *Arabidopsis*. SIF Cytoscape formation in Data S1. (d) Sub-interaction network of *Arabidopsis* pistil. .xlse file in Data S5. (e) Distribution of enzymes in maize and *Arabidopsis* pistil. .xlse file in Data S3. (f) Overlap structure of GECN models of maize and *Arabidopsis*. .xlse file in Data S3 . (g) Distribution of enzymes of *Arabidopsis* pollen tube in response to pollination. .xlse file in Data S3 and Data S5. Connected pairs of nodes analyzed by NetworkAnalyzer were marked by red. Graph was generated with the Cytoscape software [75].

The construction of GECN in maize (1,826 nodes and 4,581 edges) and *Arabidopsis* (1,149 nodes and 3,227 edges) is shown in Figure 3a and 3c (described in the “Materials and Methods”). When we mapped 6,334 significant DEGs of maize silk to the biochemical pathways of a maize GECN model, we found that 704 genes encoded 398 enzymes and that the sub-interaction network of the GECN consisted of 266 nodes (enzymes) and 355 edges (reactions) (Figure 3b; Data S5). Simultaneously, when we mapped 1,648 significant DEGs of the *Arabidopsis* stigma to an *Arabidopsis* GECN model, we found that 371 genes encoded 204 enzymes and that the sub-interaction network of the GECN model had 183 enzymes and 243 reactions (Figure 3d; Data S5). We then analyzed the number of connected pairs of nodes using NetworkAnalyzer [38].

In addition, we analyzed the overlap structure of the GECN models of maize and *Arabidopsis* (Figure 3e). Maize and *Arabidopsis* share a set of 88 enzymes that show metabolic pathways that are active in the stigma during the pollen–stigma interaction. In these 88 enzymes, we could identify three predominant groups (Figure 3f). The first, and largest, group was composed 11 nodes (enzymes) and 24 edges (reactions). The other two groups were composed of 5 nodes and 4 enzymes and 4 nodes and 4 enzymes, respectively. We also identified nine groups that were composed of 2 nodes and 1 edge.

We also mapped 1,121 genes—with significant DEGs of the *Arabidopsis* pollen tube in response to pollination—to an *Arabidopsis* GECN model. There were 348 genes encoding 116 enzymes, and the sub-interaction network of the GECN model had 102 enzymes and 61 reactions (Figure 3g; Data S5).

### Cytosolic glycolysis (plants), pyruvate dehydrogenase and the TCA cycle in lily pollen tube *in vitro*—indicating that ethanol is critical for pollen tube growth

**Figure 4:**
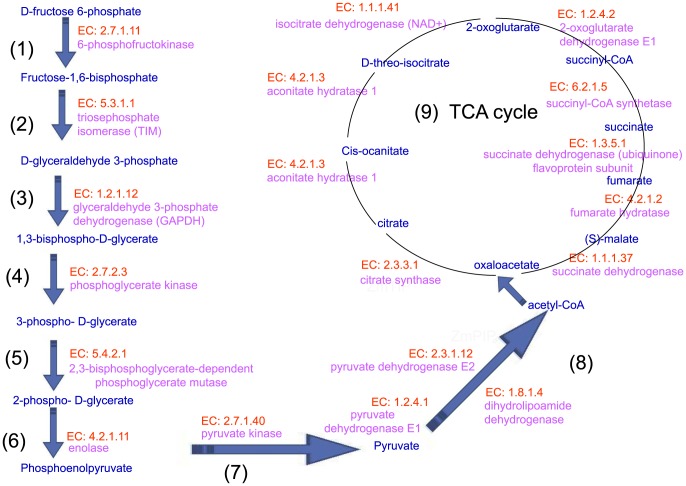
Co-expressed enzymes and consecutive steps for superpathway of cytosolic glycolysis (plants), pyruvate dehydrogenase and TCA cycle in lily pollen tube in vitro. (1) –(9) present the enzymes and substrates that catalyze consecutive steps, constructing a metabolic route. These findings support the idea that pollen germination and tube growth are high-energy-consuming processes. EC numbers of enzyme were marked by red, enzymes were marked by pink, metabolic product were marked by blue.

To provide a comprehensive overview of the metabolic pathways that are active during pollen germination and tube growth, Gerhard et al. [7] investigated the metabolome of the pollen (tube) of lily (*Lilium longiflorum*) *in vitro*. Focusing on the time-dependent changes in proteins related to carbohydrate and energy metabolism, these authors simultaneously determined more than 100 different metabolites by gas chromatography coupled with mass spectrometry. In our study, a multi-omics network analysis identified key enzymes that catalyze consecutive steps, constructing a metabolic route for a superpathway of cytosolic glycolysis (plants), pyruvate dehydrogenase and the TCA cycle in the pollen tube (Figure 4). These findings support the idea that pollen germination and tube growth are high-energy-consuming processes.

Early studies have identified the pyruvate dehydrogenase bypass by which pollen grains produce ethanol to support the TCA cycle and lipid biosynthesis [40], [41], [42]. Gerhard et al. [7] found that the inhibition of the mitochondrial electron transport chain by antimycin A resulted in an immediate production of ethanol and a fast rearrangement of metabolic pathways, whereas ethanol fermentation compensated for the reduced ATP production. Both enzymes of this bypass, pyruvate decarboxylase and alcohol dehydrogenase—were monitored. Thus, ethanol plays a critical role in pollen tube growth.

### Ethanol degradation and TCA cycle variation in the *Arabidopsis* pollen tube in response to pollination

**Figure 5:**
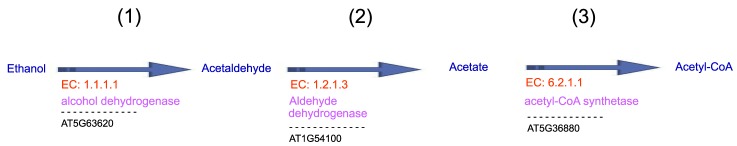
Consecutive steps of ethanol degradation II of *Arabidopsis* pollen tube in response to pollination. (1)–(3) present the significant DEGs that encoded the enzymes, enzymes and substrates that catalyze consecutive steps, thereby constructing a metabolic route for ethanol degradation II of the *Arabidopsis* pollen tube in response to pollination. EC numbers of enzyme were marked by red, enzymes were marked by pink, metabolic product were marked by blue, significant DEGs encoded enzymes were marked by black.

**Figure 6:**
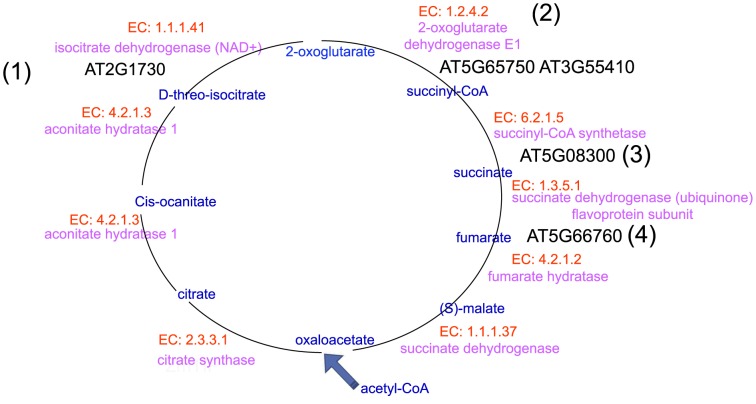
Consecutive steps of TCA cycle variation of *Arabidopsis* pollen tube in response to pollination. (1)–(4) present the significant DEGs that encoded the enzymes, enzymes and substrates that catalyze consecutive steps, thereby constructing a metabolic route for TCA cycle variation of the *Arabidopsis* pollen tube in response to pollination. EC numbers of enzyme were marked by red, enzymes were marked by pink, metabolic product were marked by blue, significant DEGs encoded enzymes were marked by black.

Our analysis identified significant DEGs that encoded co-expressed enzymes that catalyze consecutive steps, thereby constructing a metabolic route for ethanol degradation II (Figure 5) and TCA cycle variation (Figure 6) of the *Arabidopsis* pollen tube in response to pollination.

Shown in Figure 5:

1. AT5G63620 encodes alcohol dehydrogenase (EC 1.1.1.1), which catalyzes the conversion of ethanol to acetaldehyde;
2. AT1G54100 encodes aldehyde dehydrogenase (EC 1.2.1.3), which catalyzes the conversion of acetaldehyde to acetate;
3. AT5G36880 encodes acetyl-CoA synthetase (EC 6.2.1.1), which catalyzes the conversion of acetate to acetyl-CoA.

Shown in Figure 6:

1. AT2G17130 encodes isocitrate dehydrogenase (NAD+) (EC 1.1.1.41), which catalyzes the conversion of D-threo-isocitrate to 2-oxoglutarate;
2. AT5G65750 and AT3G55410 encode 2-oxoglutarate dehydrogenase E1 (EC 1.2.4.2), which catalyzes the conversion of 2-oxoglutarate to succinyl-CoA;
3. AT5G08300 encodes succinyl-CoA synthetase (EC 6.2.1.5), which catalyzes the conversion of succinyl-CoA to succinate;
4. AT5G66760 encodes succinate dehydrogenase (ubiquinone) flavoprotein subunit (EC 1.3.5.1), which catalyzes the conversion of succinate to fumarate.

Thus, these data support the idea that ethanol would be incorporated into the pollen tube's TCA cycle, leading to enhanced ATP production for facilitating pollen tube growth.

### Activation of glycolysis-related metabolic routes in *Arabidopsis* and maize stigmas to provide the fast-growing pollen tube with high-energy nutrients (alcohol/ethanol)

**Figure 7:**
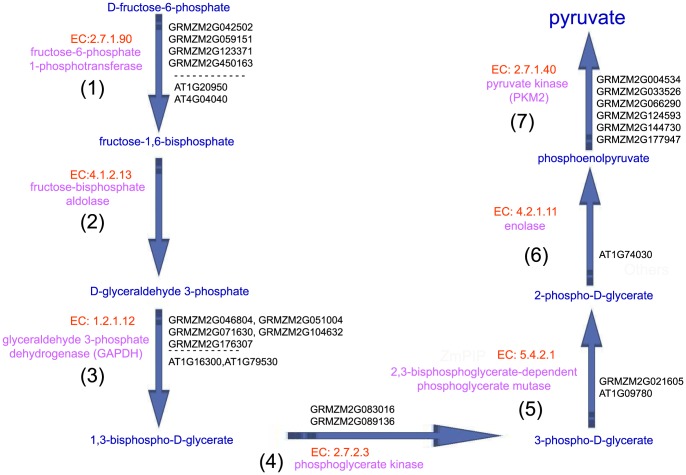
Consecutive steps of glycolysis IV pathways in *Arabidopsis* and maize stigmas. (1)–(7) present the significant DEGs that encoded the enzymes, enzymes and substrates that catalyze consecutive steps, thereby constructing a metabolic route for glycolysis IV (plant cytosol) that produce high-energy nutrients (ethanol) in *Arabidopsis* and maize stigmas during pollination. EC numbers of enzyme were marked by red, enzymes were marked by pink, metabolic product were marked by blue, significant DEGs encoded enzymes were marked by black.

Pollen tubes are the fastest growing plant cells known. This rapid growth puts great demands on energy production [43]. To fulfill these cytological requirements, protein biosynthesis and metabolic pathways in the stigma are activated to provide the fast-growing pollen tube with materials and energy [7]. As shown in Figure 7, our multi-omics network analyses show an increasing trend of the expression level of genes that encode the co-expressed enzymes and consecutive steps for glycolysis IV (plant cytosol) and the key enzymes that produce high-energy nutrients (ethanol) in *Arabidopsis* and maize stigmas during pollination.

1. Four maize genes (GRMZM2G042502, GRMZM2G059151, GRMZM2G123371 and GRMZM2G450163) and two *Arabidopsis* genes (AT1G20950 and AT4G04040) encode fructose-6-phosphate 1-phosphotransferase (EC 2.7.1.90), which catalyzes the conversion of D-fructose-6-phosphate to fructose-1,6-bisphosphate, as the initial reactions of glycolysis IV (plant cytosol).
2. In the second steps of glycolysis IV (plant cytosol), our multi-omics network analyses showed no differentially expressed maize genes or *Arabidopsis* genes encoding fructose-bisphosphate aldolase (EC 4.1.2.13), which catalyzes the conversion of fructose-1,6-bisphosphate to D-glyceraldehyde 3-phosphate. We surmise that fructose-bisphosphate aldolase may result from the cellular degradative process known as “autophagy”.
3. Five maize genes (GRMZM2G046804, GRMZM2G051004, GRMZM2G071630, GRMZM2G104632 and GRMZM2G176307) and two *Arabidopsis* genes (AT1G16300 and AT1G79530) encode glyceraldehyde-3-phosphate dehydrogenase (GAPDH) (EC 1.2.1.12), which catalyzes the conversion of D-glyceraldehyde 3-phosphate to 1,3-bisphospho-D-glycerate.
4. Two maize genes (GRMZM2G083016 and GRMZM2G089136) encode phosphoglycerate kinase (EC 2.7.2.3), which catalyzes the conversion of 1,3-bisphospho-D-glycerate to 3-phospho-D-glycerate. In particular, our analysis only identified the genes that encoded phosphoglycerate kinase, which showed significant changes in the level of expression in the maize stigma, but not in the *Arabidopsis* stigma, in response to pollination. It is possible that digital expression profiling using RNA-seq showed increased sensitivity, specificity and accuracy relative to the microarray platform. However, after re-checking the source datasets of the expression of these genes, we did find that the expression of these genes did not significantly change.
5. GRMZM2G021605 and AT1G09780 encode 2,3-bisphosphoglycerate-dependent phosphoglycerate mutase (EC 5.4.2.1), which catalyzes the conversion of 3-phospho-D-glycerate to 2-phospho-D-glycerate.
6. Our analysis only identified that AT1G74030 encodes enolase (EC 4.2.1.11), which catalyzes the conversion of 2-phospho-D-glycerate to phosphoenolpyruvate.
7. Six maize genes (GRMZM2G004534, GRMZM2G033526, GRMZM2G066290, GRMZM2G124593, GRMZM2G144730 and GRMZM2G177947) encode pyruvate kinase (PKM2) (EC 2.7.1.40), which catalyzes the conversion of phosphoenolpyruvate into pyruvate, as the end reactions of glycolysis IV (plant cytosol).

Interestingly, our analysis also identified the key enzymes that produce high-energy nutrients (ethanol). First, four maize genes (GRMZM2G014193, GRMZM2G073860, GRMZM2G134054 and GRMZM2G174549) and three *Arabidopsis* genes (AT1G04040, AT3G17790 and AT3G52820) encode acid phosphatase (EC 3.1.3.2), which catalyzes the conversion of phosphate monoester to ethanol. Second, four maize genes (GRMZM2G013324, GRMZM2G018416, GRMZM2G021482 and GRMZM2G060194) and two *Arabidopsis* genes (AT5G58050 and AT5G58170) encode glycerophosphodiester phosphodiesterase (EC 3.1.4.46), which catalyzes the conversion of glycerophosphodiester to ethanol. Thus, these data support the idea that when the pollen tube grows into the extracellular matrix in the stigma secretory zone after germination, the stigma may indeed be activated to produce a metabolic response. Indeed, glycolysis-related metabolic routes are activated: sugars are converted into pyruvate, which can then be further metabolized to high-energy nutrients (alcohol/ethanol). These nutrients can then be transported to the pollen tube.

### Co-expressed enzymes and consecutive steps for kaempferol biosynthesis

**Figure 8:**
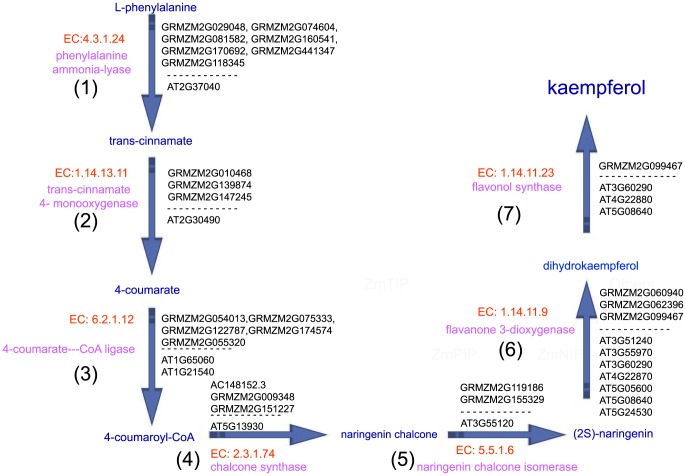
Consecutive steps of kaempferol biosynthesis pathways in *Arabidopsis* and maize stigmas. (1)–(7) present the significant DEGs that encoded the enzymes, enzymes and substrates that catalyze consecutive steps, thereby constructing a metabolic route for kaempferol biosynthesis from L-phenylalanine in *Arabidopsis* and maize stigmas. EC numbers of enzyme were marked by red, enzymes were marked by pink, metabolic product were marked by blue, significant DEGs encoded enzymes were marked by black.

Studies have shown that kaempferol, which is supplied by the pollen or stigma, is essential for pollen germination and for tube growth in petunia [44], [45]. Our analysis identified seven enzymes that catalyze consecutive steps, constructing a metabolic route for kaempferol biosynthesis from L-phenylalanine in *Arabidopsis* and maize stigmas (Figure 8).

1. Seven maize genes (GRMZM2G029048, GRMZM2G074604, GRMZM2G081582, GRMZM2G118345, GRMZM2G160541, GRMZM2G170692 and GRMZM2G441347) and one *Arabidopsis* gene (AT2G37040) encode phenylalanine ammonia-lyase (EC 4.3.1.24), which catalyzes the phenylalanine ammonia-lyase pathways as initial reactions of phenylpropanoid metabolism. Furthermore, based on the Cell Wall Genomics database (http://cellwall.genomics.purdue.edu/), GRMZM2G118345 was involved in cell-wall-related pathways for substrate generation.
2. Three maize genes (GRMZM2G010468, GRMZM2G139874 and GRMZM2G147245) and one *Arabidopsis* gene (AT2G30490) encode trans-cinnamate 4- monooxygenase (EC 1.14.13.11), which catalyzes phenylpropanoid metabolism. GRMZM2G139874 and GRMZM2G147245 are also involved in cell-wall-related oxidation-reduction pathways for substrate generation (GO:0055114).
3. Five maize genes (GRMZM2G054013, GRMZM2G055320, GRMZM2G075333, GRMZM2G122787 and GRMZM2G174574) and two *Arabidopsis* genes (AT1G65060 and AT1G21540) encode 4-coumarate:—CoA ligase (EC 6.2.1.12), which is involved in the last step of the general phenylpropanoid pathway.
4. Three maize genes (AC148152. 3, GRMZM2G009348 and GRMZM2G151227) and one *Arabidopsis* gene (AT5G13930) encode chalcone synthase (EC 2.3.1.74), which catalyzes flavonoid biosynthesis; chalcone synthase is a key enzyme involved in the biosynthesis of flavonoids.
5. GRMZM2G119186, GRMZM2G155329 and AT3G55120 encode naringenin chalcone isomerase (EC 5.5.1.6), which catalyzes the conversion of chalcones to flavanones.
6. Three maize genes (GRMZM2G060940, GRMZM2G062396 and GRMZM2G099467) and seven *Arabidopsis* genes (AT3G51240, AT3G55970, AT3G60290, AT4G22870, AT5G05600, AT5G08640 and AT5G24530) encode flavanone 3-dioxygenase (EC 1.14.11.9), which is simultaneously expressed with chalcone synthase and chalcone isomerases, thereby catalyzing flavonoid biosynthesis. AT3G60290, AT5G05600 and AT4G22870 are 2-oxoglutarate (2OG)- and Fe(II)-dependent oxygenase superfamily proteins.
7. GRMZM2G099467 and AT3G60290, AT4G22880, AT5G08640 encode flavonol synthase (EC 1.14.11.23), which catalyzes the conversion of dihydroflavonols to flavonols.

### Co-expression of L-rhamnose and the kaempferol glucoside biosynthesis pathway

**Figure 9:**
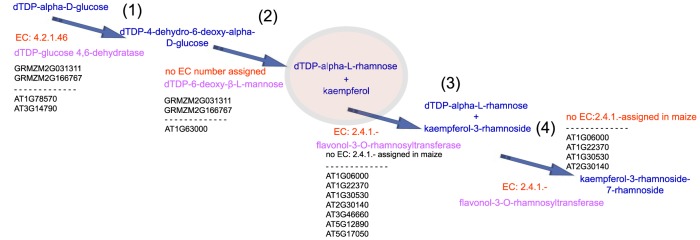
Cooperating of kaempferol and dTDP-alpha-L-rhamnose biosynthesis. (1)–(4) present the significant DEGs that encoded the enzymes, enzymes and substrates that catalyze consecutive steps, thereby constructing a metabolic route that the cooperation between kaempferol and dTDP-alpha-L-rhamnose may be involved in the regulation of cell walls in the stigmatic secretory zone and the TT. EC numbers of enzyme were marked by red, enzymes were marked by pink, metabolic product were marked by blue, significant DEGs encoded enzymes were marked by black.

As shown in Figure 9, our results reveal that many significant DEGs encode enzymes that are involved in the UDP-L-rhamnose and kaempferol glucoside biosynthesis pathway.

1. GRMZM2G031311, GRMZM2G166767 and AT1G78570 and AT3G14790 encode dTDP-glucose 4, 6-dehydratase (EC 4.2.1.46), which catalyzes the conversion of dTDP-alpha-D-glucose to dTDP-4-dehydro-6-deoxy-alpha-D-glucose, which is involved in the biosynthesis of rhamnose, a major monosaccharide component of pectin. Additionally, AT3G14790 is involved in the cell-wall-related pathways of nucleotide-sugar interconversion pathways.
2. GRMZM2G166767, GRMZM2G031311 and AT1G63000 encode enzymes that catalyze the conversion of dTDP-4-dehydro-6-deoxy-alpha-D-glucose to dTDP-alpha-L-rhamnose, which is involved in the dTDP-L-rhamnose biosynthesis II biosynthesis. GRMZM2G166767, GRMZM2G031311 and AT1G63000 are NRS/ER genes, and no EC number was assigned in the GECN models of maize and *Arabidopsis*.
3. Seven *Arabidopsis* genes (AT1G06000, AT1G22370, AT1G30530, AT2G30140, AT3G46660, AT5G12890 and AT5G17050) encode flavonol-3-O-rhamnosyltransferase (EC 2.4.1.-), which catalyzes the conversion of kaempferol and dTDP-alpha-L-rhamnose to kaempferol-3-rhamnoside, which is involved in one step of the general kaempferol glucoside biosynthesis pathway. Because there are missing enzymes (EC:2.4.1.-) in the maize genome-scale metabolic network, significant DEGs that encoded enzymes (EC: 2.4.1.-) in maize are not defined.
4. Flavonol-3-O-rhamnosyltransferase (EC: 2.4.1.-) catalyzes the conversion of kaempferol-3-rhamnoside and dTDP-alpha-L-rhamnose to kaempferol-3-rhamnoside-7-rhamnoside.

Early studies showed that L-rhamnose is an important constituent of pectic polysaccharides, a major component of cell walls. The inhibition of L-rhamnose biosynthesis leads to cell wall integrity defects [59], [60], [61]. Thus, it is possible that the cooperation between kaempferol and dTDP-alpha-L-rhamnose may be involved in the regulation of cell walls in the stigmatic secretory zone and the TT.

### Significant DEGs encoding cell wall-related proteins

Based on the Cell Wall Genomics database (http://cellwall.genomics.purdue.edu/), we found that there are many genes related to cell walls that showed significant changes at the expression level in *Arabidopsis* and maize stigmas in response to pollination. First, we identified 34 maize and 27 *Arabidopsis* genes that encoded glycosylphosphatidylinositol (GPI)-anchored proteins related to signaling and the response of the cell wall (**Data S6**). Then, we identified other genes involved in cell wall regulation, which encode cellulose synthases, lignin assembly and modification-related proteins, glycosyl transferase family proteins, expansins, polygalacturonases, glycoside hydrolase family proteins, pectin methyl esterases, fasciclin-like AGP group proteins, pectate lyases and arabinogalactan-proteins (AGPs) (**Data S6**). For example, AT3G14790 encodes dTDP-glucose 4, 6-dehydratase (EC 4.2.1.46), which is involved not only in the cell-wall-related nucleotide-sugar interconversion pathway but also in the biosynthesis of rhamnose. Furthermore, based on the proteome-wide binary protein–protein interaction map of *Arabidopsis*, we also found that five significant DEGs—AT5G14180 (encoding glycosylphosphatidylinositol (GPI)-anchored protein), AT5G03760 (cellulose synthase-like genes), AT4G30290 (encoding xyloglucan endotransglucosylase/hydrolases), AT5G44830 (encoding polygalacturonases, or “PGases”) and AT2G01630 (glycoside hydrolase family),—had significant relations with dTDP-glucose 4, 6-dehydratase (EC 4.2.1.46), which is encoded by AT3G14790 and is involved in the biosynthesis of rhamnose. In addition, expansins are a family of non-enzymatic proteins that function in cell wall loosening [46]. Our results reveal that there were 14 genes in maize and 10 genes in *Arabidopsis* encoding expansins that showed significant expression changes in stigmas in response to pollination (**Data S6**). Thus, it is possible that these proteins, cooperating with kaempferol and with L-rhamnose, play important roles in cell wall remodeling or in mediating cell-to-cell signaling reductions, which promote pollen tube growth through the stigma and style.

### Co-expressed enzymes and consecutive steps in the phosphatidic acid (PA) biosynthesis pathway

**Figure 10:**
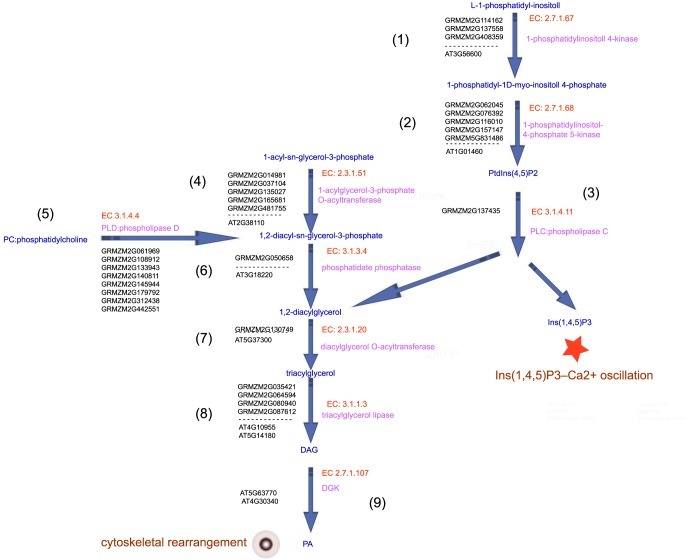
Consecutive enzymes and metabolic routes required for the synthesis of PA in maize and *Arabidopsis*. (1)–(9) present the significant DEGs that encoded the enzymes, enzymes and substrates that catalyze consecutive steps, thereby constructing a metabolic route of production of PA in *Arabidopsis* and maize stigmas. EC numbers of enzyme were marked by red, enzymes were marked by pink, metabolic product were marked by blue, significant DEGs encoded enzymes were marked by black.

Two major, distinct signaling pathways contribute to the production of PA. One pathway is a cleavage production of structural phospholipids catalyzed by phospholipase D (PLD); the other pathway is a sequence of reactions catalyzed by phospholipase C (PLC) and diacylglycerol kinase (DGK) [47], [48]. Then, the phosphorylation by phosphatidyl inositol-4,5-bisphosphate [PtdIns(4,5)P2] is hydrolyzed by PLC, which results in the production of two important second messenger molecules, D-myo-inositol-1,4,5-trisphosphate [Ins(1,4,5)P3] and diacylglycerol (DAG) [49], [50], [51]. Our analysis identified the major, distinct metabolic pathways that contribute to the production of PA in *Arabidopsis* and maize stigmas (Figure 10).

1. Three maize genes (GRMZM2G114162, GRMZM2G137558 and GRMZM2G408359) and one *Arabidopsis* gene (AT3G56600) encode phosphoinositide 4-kinase (EC 2.7.1.67), which catalyzes the conversion of L-1-phosphatidyl-inositol to 1-phosphatidyl-1D-myo-inositol 4-phosphate.
2. Five maize genes (GRMZM2G062045, GRMZM2G076392, GRMZM2G116010, GRMZM2G157147 and GRMZM5G831486) and one *Arabidopsis* gene (AT1G01460) encode phosphoinositide phosphate 5-kinase (EC 2.7.1.68), which catalyzes the conversion of 1-phosphatidyl-1D-myo-inositol 4-phosphate to PtdIns(4,5)P2.
3. GRMZM2G137435 encodes PLC (EC 3.1.4.11), which catalyzes the conversion of PtdIns(4,5)P2 to 1,2-diacylglycerol and Ins(1,4,5)P3. In particular, our analysis only identified genes encoding PLC that showed significant expression changes in the maize stigma, but not in the *Arabidopsis* stigma, in response to pollination. It is possible that the digital expression profiling that used RNA-seq showed increased sensitivity, specificity and accuracy relative to the microarray platform.
4. Five maize genes (GRMZM2G014981, GRMZM2G037104, GRMZM2G135027, GRMZM2G165681 and GRMZM2G481755) and one *Arabidopsis* gene (AT2G38110) encode 1-acylglycerol-3-phosphate O-acyltransferase (EC 2.3.1.51), which catalyzes the conversion of 1-acyl-sn-glycerol-3-phosphate to 1,2-diacyl-sn-glycerol-3-phosphate.
5. Eight maize genes (GRMZM2G061969, GRMZM2G108912, GRMZM2G133943, GRMZM2G140811, GRMZM2G145944, GRMZM2G179792, GRMZM2G312438 and GRMZM2G442551) encode PLD (EC 3.1.4.4), which catalyzes the conversion of phosphatidylcholine to 1,2-diacyl-sn-glycerol-3-phosphate. In particular, because the accuracy of the microarray platform is lower compared with the RNA-seq, our analysis only identified genes encoding PLD that showed significant expression changes in the maize stigma but not in the *Arabidopsis* stigma, in response to pollination.
6. GRMZM2G050658 and AT3G18220 encode phosphatidate phosphatase (EC 3.1.3.4), which catalyzes the conversion of 1, 2-diacyl-sn-glycerol-3-phosphate to 1,2-diacylglycerol.
7. GRMZM2G130749 and AT5G37300 encode diacylglycerol O-acyltransferase (EC 2.3.1.20), which catalyzes the conversion of 1, 2-diacylglycerol to triacylglycerol.
8. Four maize genes (GRMZM2G035421, GRMZM2G064594, GRMZM2G080940 and GRMZM2G087612) and two *Arabidopsis* genes (AT4G10955 and AT5G14180) encode triacylglycerol lipase (EC 3.1.1.3), which catalyzes the conversion of triacylglycerol into DAG.
9. Because there is no information concerning DGK (EC 2.7.1.107) in CornCyc 2.0, we did not identify any genes encoding DGK that were involved in the conversion of DAG to PA in the maize stigma. However, our analysis identified that AT5G63770 and AT4G30340 encode DGK (EC 2.7.1.107), which is involved in the conversion of DAG to PA, and showed significant expression changes in the *Arabidopsis* stigmas in response to pollination.

### Co-expressed enzymes and consecutive steps in the glutamate degradation IV pathways for GABA biosynthesis

**Figure 11:**
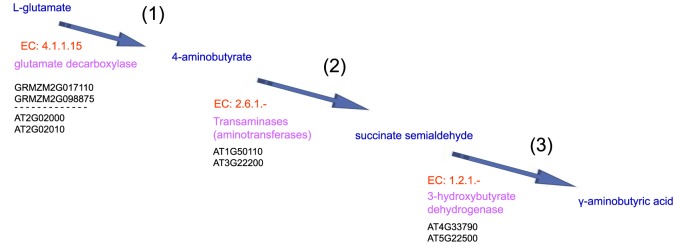
Consecutive steps of glutamate degradation IV pathways in *Arabidopsis* and maize stigmas. (1)–(3) present the significant DEGs that encoded the enzymes, enzymes and substrates that catalyze consecutive steps, thereby constructing a metabolic route for glutamate degradation IV in *Arabidopsis* and maize stigmas. EC numbers of enzyme were marked by red, enzymes were marked by pink, metabolic product were marked by blue, significant DEGs encoded enzymes were marked by black.

GABA is primarily metabolized via a short pathway termed “the GABA shunt”, which is composed of three enzymes. Our analysis identified the key enzymes that catalyze consecutive steps, thereby constructing a metabolic route for glutamate degradation IV in *Arabidopsis* and maize stigmas (Figure 11).

1. Two maize genes (GRMZM2G017110 and GRMZM2G098875) and two *Arabidopsis* genes (AT2G02000 and AT2G02010) encode glutamate decarboxylase (EC 4.1.1.15), which catalyzes the conversion of L-glutamate to 4-aminobutyrate.
2. Two *Arabidopsis* genes (AT1G50110 and AT3G22200) encode tyrosine aminotransferase (EC: 2.6.1.-), which catalyzes the conversion of 4-aminobutyrate to succinate semialdehyde.
3. Two *Arabidopsis* genes (AT4G33790 and AT5G22500) encode 3-hydroxybutyrate dehydrogenase (EC 1.2.1.-), which catalyzes the conversion of succinate semialdehyde to GABA.

## Discussion

### Inferred pattern of pollen tube-stigma ethanol coupling during pollen tube elongation in the TT of the pistil

**Figure 12:**
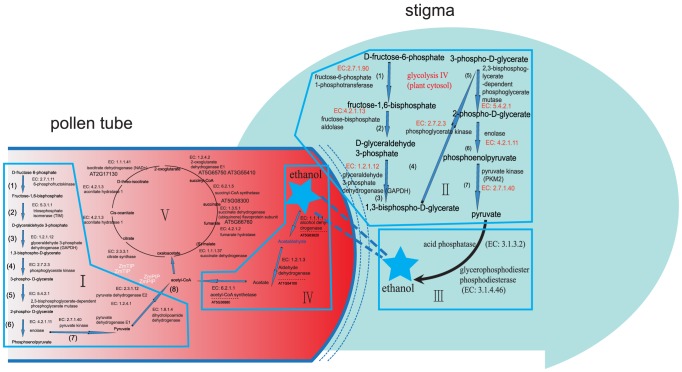
Schematic representation of the inferred pattern of pollen tube-stigma ethanol coupling when pollen tube elongation in the TT of the pistil. (i) Pollen germination and tube growth are high-energy-consuming processes. (ii) When the pollen tube elongates in the TT of the pistil, pollen tube elongation triggers the mobilization of energy from glycolysis in TT cells of the pistil, which are activated to convert sugars into pyruvate. (iii) Pyruvates are further metabolized to high-energy nutrients (alcohol/ethanol), which are secreted as energy-rich metabolites (ethanol) that can then be taken up by the pollen tube.(iv) High-energy nutrients (alcohol/ethanol) are further metabolized to acetyl-CoA by ethanol degradation II in the pollen tube.(v) Acetyl-CoA is incorporated into the pollen tube's TCA cycle variation, leading to enhanced ATP production for facilitating pollen tube growth.

Researchers in cancer cells have described that cancer cells preferentially import and utilize the lactate produced by their neighbors as their main energy source [72], [73]. In neuron-glia metabolic coupling, astrocytes undergo aerobic glycolysis, secrete energy-rich metabolites (pyruvate and lactate), and neurons then take up these metabolites and use them in the neuronal TCA cycle to generate high amounts of ATP [74]. It has been reported that chronic and acute alcohol exposure decreases cerebral glucose metabolism and increases acetate oxidation [40], [41]. Based on the mechanism from the cell-cell interaction in neuron-glia metabolic coupling and cancer cells. As shown in Figure 12, we considered the inferred pattern of pollen tube-stigma ethanol coupling. Pollen tube-stigma ethanol coupling provides the first account, to our knowledge, of the formulation of a metabolic coupling model in a plant.

1. Pollen germination and tube growth are high-energy-consuming processes. In particular, the superpathway, which is composed of cytosolic glycolysis (plants), pyruvate dehydrogenase, the TCA cycle, and ethanol is critical for pollen tube growth.
2. When the pollen tube elongates in the TT of the pistil, pollen tube elongation triggers the mobilization of energy from glycolysis in TT cells of the pistil, which are activated to convert sugars into pyruvate.
3. Pyruvates are further metabolized to high-energy nutrients (alcohol/ethanol), which are secreted as energy-rich metabolites (ethanol) that can then be taken up by the pollen tube.
4. High-energy nutrients (alcohol/ethanol) are further metabolized to acetyl-CoA by ethanol degradation II in the pollen tube.
5. Acetyl-CoA is incorporated into the pollen tube's TCA cycle variation, leading to enhanced ATP production for facilitating pollen tube growth.

### Cooperation of kaempferol, L-rhamnose and cell-wall-related proteins may be involved in cell wall loosening in the stigmatic secretory zone and the TT

The pollen tube grows into the extracellular matrix in the stigma secretory zone after germination, and cell wall loosening of the stigma is important for pollen tube growth [52]. Flavonols are essential for pollen germination and tube growth, during which flavonols can be supplied by either the pollen or stigma at pollination [53]–[58]. Kaempferol, which is the major component of flavonoids, increased to a detectable level in the outer cell layers and exudate of petunia stigmas when petunia pollen tubes grew into the TT [44], [45]. In addition, early studies showed that L-rhamnose is an important component of cell walls and that the inhibition of L-rhamnose biosynthesis leads to cell wall integrity defects [59], [60], [61]. However, the mechanisms of kaempferol and L-rhamnose for manipulating pollen tube growth in the stigma are unknown. In our study, we first identified seven enzymes and metabolic routes that catalyzed the consecutive steps of kaempferol biosynthesis in *Arabidopsis* and maize stigmas during pollen tube elongation in the TT of the pistil. Second, our results also indicated that there is cooperation between kaempferol and TDP-alpha-L-rhamnose in the regulation of pollen tube growth in the pistil tissue. Furthermore, many genes encoding proteins related to the cell wall showed significant changes in *Arabidopsis* and maize stigmas during pollen tube elongation in the TT of the pistil, and these proteins (enzymes) catalyzed kaempferol biosynthesis based on the proteome-wide binary protein–protein interaction map of *Arabidopsis*. For example, in kaempferol biosynthesis from L-phenylalanine of *Arabidopsis* and maize stigmas, GRMZM2G118345, which encodes phenylalanine ammonia-lyase (EC 4.3.1.24), and GRMZM2G139874 and GRMZM2G147245, which encode cinnamate-4-monooxygenase (EC:1.14.13.11), were also implicated in cell -wall-related processes according to the Cell Wall Genomics database(http://cellwall.genomics.purdue.edu/) [accessed 23/01/14]. In addition, expansins are a family of non-enzymatic proteins that function in cell wall loosening [46]. Our results reveal that there are 14 genes encoding expansins in maize and 10 genes in *Arabidopsis* that showed significant expression changes in stigmas during pollen tube elongation in the TT of the pistil. We suggested that the cooperation of kaempferol, dTDP-alpha-L-rhamnose and cell-wall-related proteins appear to be crucial for regulating cell wall remodeling in the stigmatic secretory zone and the TT. The cooperation of these proteins tends to play a role in regulating pollen tube growth by loosening the cell wall in the stigmatic secretory zone and the TT.

### PA-mediated Ca^2+^ oscillations and cytoskeleton remodeling may be exploited by the stigma to facilitate pollen tube growth

Phosphatidic acid (PA), which is a lipid-signaling mediator involved in various cellular processes, has been shown to play a critical role in the regulation of the tip growth of the pollen tube and the root hair [47]. Potocky et al. [62] first reported the importance of PA catalyzed by PLD in the polarized expansion of plant cells. Then, Monteiro et al. [63] showed that PtdIns(4,5)P2 and Ins(1,4,5)P3, together with PA, play a vital role in the regulation of cytoplasmic [Ca^2+^] levels, endo/exocytosis, and vesicular trafficking in the apex of pollen tubes. Next, numerical detailed studies showed that PLC is targeted to the plasma membrane laterally at the pollen tube tip and maintains the apical domain enriched in PtdIns(4,5)P2 and DAG, which are required for polar cell growth [64], [65], [66]. Recently, using tobacco pollen tubes as a model, Pleskot et al. [67] addressed the importance of PLD, DGKs and lipid phosphate phosphatases involved in PA production and degradation during pollen tube growth. The phosphatidylcholine system is an important component for the regulation of Ca^2+^ dynamics, in which the hydrolysis of PtdIns(4,5)P2 produces two messengers: Ins(1,4,5)P3 and DAG [47]. Depending on the calcium concentration, PLC is able to hydrolyze different substrates. At low (micromolar) Ca^2+^ concentrations, PtdIns(4,5)P2 is hydrolyzed, whereas at higher (millimolar) Ca^2+^ concentrations, PLC preferentially uses phosphatidylinositol phosphate (PIP) as the substrate. Numerical detailed studies have shown that PtdIns(4,5)P2 leads to increased [Ca^2+^], whereas Ins(1,4,5)P3 causes a transient [Ca^2+^] increase of similar magnitude [64], [65], [66]. In addition, Monteiro et al. [63] demonstrated that PtdIns(4,5)P2 and Ins(1,4,5)P3 signaling consists of a feedback loop from the cytosol to the plasma membrane through a multiple pathway system that involves the regulation of [Ca^2+^] levels, endo/exocytosis, actin cytoskeleton dynamics, and vesicular trafficking. However, the mechanisms of PA and their derived messenger molecules in the stigma for manipulating pollen tube growth are unknown. As shown in Figure 5, our results revealed that phosphorylation of 1-phosphatidyl-1D-myo-inositol 4-phosphate was performed by specific phosphoinositide kinases, including phosphoinositide 4-kinase and phosphoinositide phosphate 5-kinase, to generate PtdIns(4,5)P2, which is then hydrolyzed by PLC, resulting in the production of Ins(1,4,5)P3 and DAG (1,2-diacylglycerol →triacylglycerol→DAG). Ins(1,4,5)P3 plays a role in regulating the oscillation of [Ca^2+^].

In plants, both the actin and microtubular cytoskeletons can be regulated by phospholipids and can participate in regulating the tip growth of the root hair and the pollen tube. PtdIns(4,5)P2, Ins(1,4,5)P3, PA and the related phosphoinositide 3-kinase, phosphoinositide 4-kinase and phosphoinositide phosphate 5-kinase are involved in the organization of actin filaments [47]. PtdIns(4,5)P2 controls the dynamic organization of filamentous actin through the regulation of profilin, which is a G-actin (globular actin)-binding protein, and actin remodeling in root hairs and pollen tubes [68]. Our analysis identified the major, distinct signaling pathways that contribute to the production of PA in *Arabidopsis* and maize stigmas when pollen tubes elongate in the TT of the pistil. The key enzymes involved in PA biosynthesis were identified in our analysis and included DAG, DGK, phosphatidylcholine, PtdIns(4,5)P2, Ins(1,4,5)P3, DAG and PA. Thus it is possible that PA-mediated cytoskeleton and vesicle trafficking are exploited by the stigma to provide the large amount of material required for the pollen tube tip growth of the plasma membrane and the cell wall.

### Glutamate degradation IV activation provides the fast-growing pollen tube with a GABA signal

GABA is a 4-carbon non-protein amino acid found in a wide range of organisms. Recently, genetic and genomic approaches have shown that calcium-dependent GABA signaling regulates pollen germination and polarized tube growth by affecting actin filament patterns, vesicle trafficking and the configuration and distribution of cell wall components [69], [70], [71]. Our analysis identified key enzymes that catalyze consecutive steps, thereby constructing a metabolic route for glutamate degradation IV in *Arabidopsis* and maize stigmas, which are activated to provide the fast-growing pollen tube with a GABA signal.

## Conclusions

Vascular plants are likely to exhibit similar (or identical) metabolism, the central metabolism may be conserved (or be very similar) between the species [77], [78]. Combined analysis of the maize (monocot) and Arabidopsis (dicot) reveals the general mechanism of communication between male and female gametes. Because of the difficulties in deriving qualitative or, at best, semi quantitative proteomics and metabolomic experimental data relating to male (pollen tube) and female (pistil) reproductive tissues, there is not the state-of-art dataset available for *Arabidopsis* or maize about the pollen-stigma communication.

In our study, based on the mechanism from the cell-cell interaction in neuron-glia metabolic coupling and cancer cells, we respectively reconstructed the GECN models of maize and *Arabidopsis*, and also respectively draw the conclusions for maize and Arabidopsis. Our analysis indicates the inferred pattern of pollen tube-stigma ethanol coupling. When the pollen tube elongates in the TT of the pistil, this elongation triggers the mobilization of energy from glycolysis in the TT cells of the pistil. Energy-rich metabolites (ethanol) are secreted that can be taken up by the pollen tube, where these metabolites are incorporated into the pollen tube's tricarboxylic acid (TCA) cycle, leading to enhanced ATP production for facilitating pollen tube growth. In addition, our analysis also supports the idea that the cooperation of kaempferol, dTDP-alpha-L-rhamnose and cell-wall-related proteins, PA-mediated Ca^2+^ oscillations and the cytoskeleton, and glutamate degradation IV for GABA signaling are activated in *Arabidopsis* and maize stigmas to provide the fast-growing pollen tube with the signals and materials required for pollen tube tip growth.The metabolome of pollen of lily (*Lilium longiflorum*) also support our conclusions with *Arabidopsis* and maize. In particular, the expression profiling and multi-omics network analysis and the genome-scale enzyme correlation network models (GECN) developed in this study were initiated with experimental “omics” data, followed by data analysis and data integration to determine correlations, and could provide a new platform to help us achieve a deeper understanding of the coregulation and interregulation models in plant research. However, the *in silico* analysis of genes that encode enzyme and metabolites is insufficient to define their potential roles. In the future, further support for the inferred pattern of pollen tube-stigma metabolic coupling during pollination should be provided using reverse genetic approaches, such as increasing or decreasing their transcript levels (by T-DNA insertion and/or RNAi) to elucidate their biological functions.

In this manuscript, our major concern lies in identification of active modules by the usage of molecular profiles of the metabolomic pathways in one general mechanism of communication between male and female gametes. In order to reach a deeper analyzing the global, consistent prediction of metabolic behavior, our next research work is needed by solving a constraint-based modeling optimization problem to find a steady-state metabolic flux distribution (that is, an assignment of fluxes to all the reactions in the network). Such as: Constraint-based methods for analysing metabolic networks, including the widely exploited flux balance analysis (FBA) method. Recently, using GWAS and linkage mapping analysis, Wen et al. [76]. present a comprehensive study of maize metabolism, combining genetic, metabolite and expression profiling methodologies to dissect the genetic basis of metabolic diversity in maize kernels. We will try to improve the analyzing the metabolome of maize using our computational pipeline, GWAS and linkage mapping analysis in the future.

## Supporting Information

Data S1 **The construction model of GECN in** ***Arabidopsis*** **(SIF Cytoscape formation).**
(SIF)

Data S2 **The construction model of GECN in maize (SIF Cytoscape formation).**
(SIF)

Data S3 **Significantly differentially expressed genes (DEGs) of** ***Arabidopsis*** **pollen tube and** ***Arabidopsis*** **/maize stigma in response to pollination.**
(XLSX)

Data S4 **Gene Ontology (GO) enrichment analysis of significantly differentially expressed genes (DEGs) in** ***Arabidopsis*** **pollen tube and** ***Arabidopsis*** **/maize stigma in response to pollination.**
(TXT)

Data S5 **Sub-interaction network of a genome-scale enzyme correlation networks (GECN) model for** ***Arabidopsis*** **pollen tube and** ***Arabidopsis*** **/maize stigma in response to pollination.**
(XLSX)

Data S6 **Significantly differentially expressed genes encoding protein which may function in the cell wall in** ***Arabidopsis*** **pollen tube and stigma in response to pollination.**
(DOCX)
