# Supplementary material for: Transcriptional Evidence for Inferred Pattern of Pollen Tube-Stigma Metabolic Coupling during Pollination
Source: PLoS One. 2014 Sep 12;9(9):e107046. doi: 10.1371/journal.pone.0107046 (PMC4162560; doi:10.1371/journal.pone.0107046)
Supplement: Data S6 — Significantly differentially expressed genes encoding protein which may function in the cell wall in Arabidopsis pollen tube and stigma in response to pollination. (DOCX) [file pone.0107046.s006.docx]

**Significantly differential expressed genes encoding protein which may be function in the cell wall.**

| **GENE_FAMILY** | **SUB_FAMILY** | | **GENE_NAME** | |
| --- | --- | --- | --- | --- |
| **Arabidopsis** | | | | |
| Differentiation and Secondary Wall Formation | | Lignin assembly and modification:Peroxidases | | AT1G05260,AT2G37130,AT2G38380,AT2G38390,AT3G03670,AT4G11290,  AT5G05340,AT5G19880,AT5G39580,AT5G40150,AT5G47000,AT5G64110,AT5G64120 |
| Differentiation and Secondary Wall Formation | | Lignin assembly and modification: Laccases | | AT5G48100: LAC15 ,AT1G18140:LAC1 |
| Differentiation and Secondary Wall Formation | | Glycosyl transferases  GT family | | AT1G24170 AT1G70090 AT3G06260 AT4G02130 AT4G16600  AT4G16600 AT3G62720 AT1G68470 AT2G31990 |
| Differentiation and Secondary Wall Formation | | Growth modifying proteins: Expansins | | AT1G26770 AT1G69530 AT2G03090 AT2G39700 AT3G45960  AT3G45970 AT3G60570 AT4G28250 AT5G39280 AT5G39300 |
| Hydrolases | | Endo-acting lycanases  Polygalacturonases- PGases | | AT1G02790 AT2G23900 AT2G40310 AT3G06770 AT3G07820 AT3G07840 AT3G59850 AT5G44830 AT5G44840 AT5G48140 |
| Hydrolases | | Endo-acting lycanases  Glycoside hydrolase family 9 (Endo-1,4-beta-glucanase) | | AT1G19940 AT1G64390 AT2G44560 AT1G71380 AT3G43860  AT4G23560 AT4G24260 |
| Hydrolases | | Endo-acting g lycanases Glycoside hydrolase family | | AT2G01630 AT2G39640 |
| Hydrolases | | Endo-acting glycanases Glycoside hydrolase family 17 | | AT3G57260 AT4G26830 AT5G20330 AT5G20390 AT5G55180  AT5G64790 AT5G64790 |
| Hydrolases | | Esterases Pectin methyl esterases | | AT1G02810 AT2G26450 AT2G47030 AT2G47040 AT2G47550AT3G05610  AT3G10720 AT3G17060 AT3G59010 AT3G62170 AT5G07420  AT5G07430 |
| Hydrolases | | Exo-acting glycanases | | AT2G16730 AT3G52840 AT4G35010 AT5G20710 |
| Hydrolases | | Fasciclin-like AGP Group | | AT2G04780 AT2G24450 AT4G12730 AT5G55730 |
| Hydrolases | | Pectate lyases and pectin Lyases | | AT1G67750 AT2G02720 AT3G01270 AT5G09280 AT5G15110  AT5G63180 |
| Hydrolases | | Arabinogalactan-proteins (AGPs) | | AT2G22470 AT3G01700 AT3G20865 AT3G57690 AT5G14380 AT5G53250 AT5G65390 |
| Nucleotide-sugar interconversion pathways | |  | | AT1G12780 AT1G63000 AT1G78570 AT3G14790 AT4G10960  AT5G39320 AT5G44480 |
| Pathways of phenylpropanoid biosynthesis | |  | | AT1G21120 AT1G21130 AT1G65060 AT1G76470 AT1G77520  AT1G77530AT1G80820 AT2G37040 AT3G53140 AT4G37990 |
| Pathways of phenylpropanoid biosynthesis | |  | | AT1G21120 AT1G21130 AT1G65060 AT1G76470 AT1G77520  AT1G77530 AT1G80820 AT2G37040 AT3G53140 AT4G37990 |
| Polysaccharide synthases and glycosyl transferases | | Cellulose synthase-like Genes | | AT2G33100 AT3G56000 AT4G38190 AT5G03760 |
| Signaling and Response Mechanisms | | Glycosylphosphatidylinositol (GPI)-anchored proteins | | AT1G05450 AT1G08500 AT1G09790 AT1G18280 AT1G23040  AT1G24520 AT1G65240 AT1G72230 AT3G06035 AT3G07390  AT3G20580 AT3G26110 AT3G27410 AT3G52370 AT3G52370  AT4G08670 AT4G12420 AT4G28280 AT4G36010 AT5G14150  AT5G14180 AT5G15350 AT5G50660 AT5G51480 AT5G58050  AT5G58170 AT5G60950 |
| Xyloglucan endotransglucosylase/ hydrolases | |  | | AT2G14620 AT3G48580 AT4G30290 AT5G48070 |
| **Maize** | | | | |
| Assembly, Architecture, and Growth | | Pectin methyl esterases | | GRMZM2G025182 GRMZM2G128549 GRMZM2G125356 GRMZM2G141873  GRMZM2G167637 GRMZM2G162333 GRMZM2G156365 GRMZM2G160569  GRMZM2G175499 GRMZM2G431856 |
| Assembly, Architecture, and Growth | | Growth modifying proteins Expansins | | GRMZM2G021427 GRMZM2G074585 GRMZM2G073373 GRMZM2G072121 GRMZM2G095968 GRMZM2G082520 GRMZM2G105844 GRMZM2G127072  GRMZM2G154178 GRMZM2G169967 GRMZM2G342246 GRMZM2G361064  GRMZM2G368886 GRMZM2G453805 |
| Assembly, Architecture, and Growth | | Endo-acting glycanases Polygalacturonases - PGases | | GRMZM2G004435 GRMZM2G052844 GRMZM2G079263 GRMZM2G119494 GRMZM2G151755 GRMZM2G139828 GRMZM2G374375 |
| Assembly, Architecture, and Growth | | Exo-acting glycanases?-Galactosidase family 35 | | GRMZM2G038281 GRMZM2G121495 GRMZM2G130375  GRMZM2G127123 GRMZM2G178106 GRMZM2G175779  GRMZM2G417455 GRMZM2G386824 |
| Assembly, Architecture, and Growth | | Endo-acting glycanases Glycoside hydrolase family (Endo-1,4-beta-glucanase) | | GRMZM2G030850 GRMZM2G046101 GRMZM2G046459 GRMZM2G061403  GRMZM2G076049 GRMZM2G072526 GRMZM2G099101 GRMZM2G114140  GRMZM2G110735 GRMZM2G111143 GRMZM2G111324 GRMZM2G113420  GRMZM2G127117 GRMZM2G125032 GRMZM2G152638  GRMZM2G147422 GRMZM2G147849 GRMZM2G137535  GRMZM2G178025 GRMZM2G172537 GRMZM2G331566 GRMZM2G458164 |
| Assembly, Architecture, and Growth | | Hydroxyproline-rich glycoproteins (HRGPs) Arabinogalactan-proteins (AGPs) | | GRMZM2G003752 GRMZM2G011742 GRMZM2G035933 GRMZM2G065718  GRMZM2G144610 GRMZM2G421415 |
| Assembly, Architecture, and Growth | | Hydroxyproline-rich glycoproteins (HRGPs) Prolyl-4-hydroxylases | | GRMZM2G520535 GRMZM2G025867 |
| Assembly, Architecture, and Growth | | Lyases Pectate and pectin Lyases | | GRMZM2G005562 GRMZM2G080056 GRMZM2G131912 GRMZM2G364349 GRMZM2G412207 |
| Assembly, Architecture, and Growth | | yloglucan endotransglucosylase/hydrolases | | GRMZM2G004699 GRMZM2G063566 GRMZM2G060837 GRMZM2G062811 GRMZM2G110299 GRMZM2G166944 GRMZM2G180870 GRMZM2G413006 GRMZM2G392125 |
| Differentiation and Secondary Wall Formation | | Lignin assembly and modification Peroxidases | | GRMZM2G012263 GRMZM2G023840 GRMZM2G048775 GRMZM2G061230  GRMZM2G080183 GRMZM2G394500 GRMZM2G471357 GRMZM2G460406  GRMZM2G450233 GRMZM2G341934 GRMZM2G095404 GRMZM2G085967  GRMZM2G108153 GRMZM2G103342 GRMZM2G150134 GRMZM2G144648  GRMZM2G140667 |
| Differentiation and Secondary Wall Formation | | Lignin assembly and modification Laccases | | GRMZM2G169033 GRMZM2G320786 GRMZM2G132169  GRMZM2G094375 |
| Pathways of substrate generation | | Nucleotide-sugar interconversion pathways | | GRMZM2G000632 GRMZM2G007195 GRMZM2G007404 GRMZM2G031311 GRMZM2G029856 GRMZM2G040397 GRMZM2G042179 GRMZM2G038598 GRMZM2G063949 GRMZM2G072911 GRMZM2G124434 GRMZM2G110558 GRMZM2G138907 GRMZM2G166767 GRMZM2G167872 GRMZM2G170336 GRMZM2G429118 GRMZM2G381473 |
| Pathways of substrate generation | | Pathways of phenylpropanoid biosynthesis | | GRMZM2G010468 GRMZM2G035584 GRMZM2G034069 GRMZM2G034360 GRMZM2G033555 GRMZM2G029048 GRMZM2G046070 GRMZM2G050072 GRMZM2G055320 GRMZM2G054013 GRMZM2G054013 GRMZM2G074604 GRMZM2G090980  GRMZM2G081582 GRMZM2G107076 GRMZM2G107851 GRMZM2G099363 GRMZM2G118345 GRMZM2G118610 GRMZM2G122787 GRMZM2G127948 GRMZM2G147245 GRMZM2G139874 GRMZM2G138074 GRMZM2G167613 GRMZM2G160541 GRMZM2G156816 GRMZM2G156296 GRMZM2G179703 GRMZM2G170692 GRMZM2G332522 GRMZM2G441347 |
| Polysaccharide synthases and glycosyl transferases | | Callose synthase genes | | GRMZM2G022856 GRMZM2G084802 GRMZM2G111529  GRMZM2G180951 GRMZM2G341918 GRMZM2G430680 GRMZM2G465764 |
| Polysaccharide synthases and glycosyl transferases | | Cellulose synthase-like Genes:Cellulose-synthase-like genes | | GRMZM2G015886 GRMZM2G028286 GRMZM2G027794 GRMZM2G061764  GRMZM2G074792 GRMZM2G110145 GRMZM2G103972 GRMZM2G105631  GRMZM2G099088 GRMZM2G122431 GRMZM2G122277 GRMZM2G178880  GRMZM2G173759 GRMZM2G405567 |
| Polysaccharide synthases and glycosyl transferases | | Cellulose synthases | | GRMZM2G018241 GRMZM2G028353 GRMZM2G025231 GRMZM2G027723 GRMZM2G039454 GRMZM2G082580 GRMZM2G112336 GRMZM2G113137 GRMZM2G111642 GRMZM2G177631 |
| Polysaccharide synthases and glycosyl transferases | | Glycosyl transferases | | GRMZM2G000581 GRMZM2G000976 GRMZM2G002023 GRMZM2G007185 GRMZM2G015983 GRMZM2G008501 GRMZM2G036918 GRMZM2G026889 GRMZM2G023020 GRMZM2G048008 GRMZM2G045467 GRMZM2G054350 GRMZM2G056702 GRMZM2G058472 GRMZM2G059825 GRMZM2G057779 GRMZM2G080231 GRMZM2G075942 GRMZM2G075942 GRMZM2G076276 GRMZM2G103785 GRMZM2G107854 GRMZM2G115762 GRMZM2G135195 GRMZM2G127416 GRMZM2G130046 GRMZM2G149024 GRMZM2G147145 GRMZM2G144873 GRMZM2G166903 GRMZM2G165919 GRMZM2G160958 GRMZM2G386971 GRMZM2G391000 GRMZM2G448834 |
| Signaling and Response Mechanisms | | Glycosylphosphatidylinositol (GPI)-anchored protein | | GRMZM2G006377,GRMZM2G018416,GRMZM2G013324,GRMZM2G036826,GRMZM2G027825,GRMZM2G049693,GRMZM2G048194,GRMZM2G066326,GRMZM2G064605,GRMZM2G060194,GRMZM2G076225,GRMZM2G076985,GRMZM2G073465,GRMZM2G071970,GRMZM2G098298,GRMZM2G133029,GRMZM2G129064,GRMZM2G148536,GRMZM2G151589,GRMZM2G149809,GRMZM2G150256,GRMZM2G142584,GRMZM2G166281,GRMZM2G168588,GRMZM2G168115,GRMZM2G335978,GRMZM2G363813,GRMZM2G329181 GRMZM2G421463 GRMZM2G410991 GRMZM2G377215 GRMZM2G402584 GRMZM2G476523 GRMZM2G438386 |
